# Multimodal Assessment of Long-Term Memory Recall and Reinstatement in a Combined Cue and Context Fear Conditioning and Extinction Paradigm in Humans

**DOI:** 10.1371/journal.pone.0076179

**Published:** 2013-10-07

**Authors:** Jan Haaker, Tina B. Lonsdorf, Alexandra Thanellou, Raffael Kalisch

**Affiliations:** 1 Institute for Systems Neuroscience, University Medical Center Hamburg-Eppendorf (UKE), Hamburg, Germany; 2 Neuroimaging Center Mainz (NIC), Focus Program Translational Neuroscience (FTN), Johannes Gutenberg University Medical Center, Mainz, Germany; University of Akron, United States of America

## Abstract

Learning to predict danger via associative learning processes is critical for adaptive behaviour. After successful extinction, persisting fear memories often emerge as returning fear. Investigation of return of fear phenomena, e.g. reinstatement, have only recently began and to date, many critical questions with respect to reinstatement in human populations remain unresolved. Few studies have separated experimental phases in time even though increasing evidence shows that allowing for passage of time (and consolidation) between experimental phases has a major impact on the results. In addition, studies have relied on a single psychophysiological dimension only (SCRs/SCL or FPS) which hampers comparability between different studies that showed both differential or generalized return of fear following a reinstatement manipulation. In 93 participants, we used a multimodal approach (fear-potentiated startle, skin conductance responses, fear ratings to asses fear conditioning (day 1), extinction (day 2) as well as delayed memory recall and reinstatement (day 8) in a paradigm that probed contextual and cued fear intra-individually. Our findings show persistence of conditioning and extinction memory over time and demonstrate that reinstated fear responses were qualitatively different between dependent variables (subjective fear ratings, FPS, SCRs) as well as between cued and contextual CSs. While only the arousal-related measurement (SCRs) showed increasing reactions following reinstatement to the cued CSs, no evidence of reinstatement was observed for the subjective ratings and fear-related measurement (FPS). In contrast, for contextual CSs, reinstatement was evident as differential and generalized reinstatement in fear ratings as well as generally elevated physiological fear (FPS) and arousal (SCRs) related measurements to all contextual CSs (generalized non-differential reinstatement). Returning fear after reinstatement likely depends on a variety of variables (experimental design, dependent measurements) and more systematic investigations with respect to critical determinants of reinstatement in humans are required.

## Introduction

Learning to predict danger is critical for adaptive behavior in changing environments and this learning process is reflected in conditioned fear.

If an aversive event (unconditioned stimulus, US) is predicted by a discrete cue (i.e., cue conditioning), phasic fear responses are elicited, whereas the absence of a discrete danger predictor (conditioned stimuli, CSs) induces sustained anxiety responses to the global situation (i.e., context conditioning) [1]. Fear conditioning serves as a model for the etiology of anxiety disorders, and the aforementioned distinct modes of conditioning, differing with respect to the predictability of a US, have been proposed to model diverse features of anxiety disorders [2].

Moreover, learning to disregard a CS that has lost the predictive power for the US (extinction learning) also serves successful adaptation and is an important mechanism underlying behavioral treatment of pathological fears [3] as well as resilience to stress or trauma [4].

Fear conditioning and extinction induce formation of an excitatory fear (CS-US association) and an inhibitory extinction memory (CS-noUS association), respectively [5], [6]. At a later test, CS re-confrontation after successful extinction induces the retrieval of the CS-US association in parallel with the CS-no US association. The ensuing memory competition determines the degree of extinction (inhibition of the conditioned reaction [CR]) vs. fear recall (return of the CR). The processes underlying the return of fear are taken as models for relapse in clinical settings after successful therapy [3], [5], [7].

Dominance of the fear memory trace can be facilitated through contextual changes between extinction and test (renewal) [8], the mere passage of time (spontaneous recovery) [9] or unsignaled presentations of the US alone before testing (reinstatement) [10], [11].

In the present study, we focus on reinstatement as one avenue towards return of fear. A clinical example of reinstatement is the case of an individual that develops a driving phobia following serious injuries ( = US) in a car accident ( = CS). After successful cognitive-behavioural treatment of this phobia, the association between driving a car and injury may be reinstated when the same individual is injured when doing sports. As a consequence, there may be a relapse of the individuals' previous driving phobia.

In human laboratory experiments, reinstatement has so far only been investigated using cue conditioning paradigms. The above example however makes clear that fears of situations or configurations of cues (driving a car) may as well be reinstated, motivating an interest in investigating reinstatement of context CRs.

Reinstatement has first been demonstrated in animals but of note, it has primarily been tested in non-differential (single CS) conditioning paradigms [12]–[16]. Human studies, in turn, focused on differential protocols [except for 18,19].

In these studies, reinstatement of the CR was specifically observed to the previously US-predictive cue (CS+) [18], [20]–[24] while others found enhanced CRs to CS+ and the previously non-US-predictive cue (CS-, non-differential return of fear) [24]–[26]. Of note, studies observing differential return of the CR to the CS+ also, to a certain degree, observed enhanced reactions to the CS- following reinstatement [22], [23], [26]. To date, it remains unresolved which conditions determine whether reinstatement induces differential vs. generalized return of the CR. This question is important since the ability to discriminate safety cues from threat cues is negatively associated with pathological anxiety [27] and predictive of resilient responding to life stress [28]. The ability to maintain this discrimination under aversive circumstances might then be a similarly important mechanism underlying long-term remission and/or resilience.

Human studies have used skin conductance responses (SCRs)/ skin conductance level (SCL) [18], [19], [26], [29], fear potentiated startle (FPS) [21], [24] as well as fear/US expectancy ratings [20], [22], [24]–[26], [29], [30] or reaction times [20], [22], [25], [30] as CR measurements, but even within a single modality, both differential and generalized reinstatement effects have been observed. As individual studies have to date relied on a single psychophysiological dimension only (SCRs/SCL or FPS) comparability between the results of different studies is not straightforward. This makes a multimodal assessment of reinstatement desirable.

Finally, experimental phases (acquisition, extinction, reinstatement, reinstatement test) in the human studies mostly occurred on the same day, immediately following upon each other [18], [20], [22], [24]–[26], [29], [30] and in most studies reinstatement followed directly after immediate extinction [18]–[20], [22], [23], [25], [26], [29], [30].

Few studies have separated experimental phases in time. Norrholm and colleagues [21] demonstrated reinstatement after a 24 h delayed extinction and Golkar and colleagues observed less reinstatement in the immediate extinction group compared with a group undergoing 24 h delayed extinction [24]. But again, both studies have not used delayed memory tests, but have assessed reinstatement immediately after extinction. While Milad and colleagues as well as Schiller and colleagues [19], [23] used delayed memory tests 24 h after extinction, both studies have used immediate extinction in the first place.

There is increasing evidence that allowing for passage of time and thereby consolidation between the experimental phases has a major impact on the results [24], [31]. Furthermore a temporal gap between experimental phases represents a more naturalistic model for clinical relapse and facilitates translation to the results generated by animal studies.

Thus, to address these various methodological issues, we here extend an established conditioning paradigm [32]. We present cue and contextual CSs within the individual in a 3-day paradigm investigating the expression of fear and extinction memories before and after reinstatement USs one week following conditioning and delayed extinction. Thereby, we employed multimodal assessments by combining subjective ratings with arousal- (SCRs) and fear-related (fear-potentiated startle) measures.

Given the important role of the context in reinstatement, we were particularly interested in whether reinstatement effects would be observed for both cued and contextual fear and whether the type of dependent variable (arousal [SCRs] or fear-specific [FPS]) would be related to cued and contextual return of fear.

## Materials and Methods

### Overview over design

**Figure 1:**
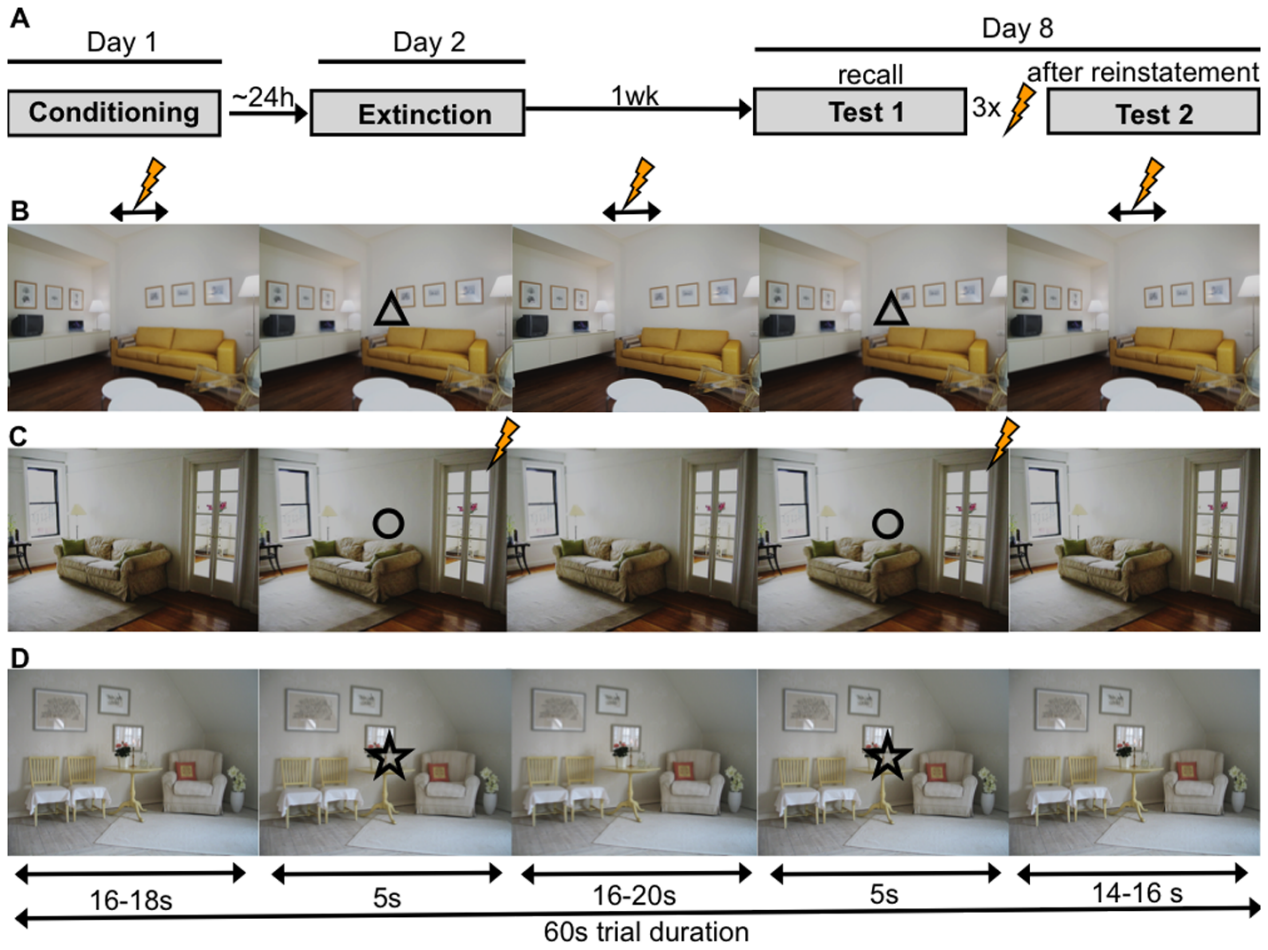
Design. Experimental timeline (**A**) and structure of trials in the unpredictable (**B**), predictable (**C**) as well as safe (**D**) trials. Shown is an example of stimulus-condition assignments. Bolt denotes US.

We used a multiple-day operationalization (see **Fig. 1A**) of combined cue- and context- conditioning (‘NPU-threat test’) proposed by Grillon and coworkers [2], [33] based on earlier animal work [34]. This paradigm allows for studying context conditioning (sustained fear) and cue conditioning (phasic fear) [35], [36] within the same experiment and subject and has previously been used in our laboratory to disentangle the neural underpinnings of cued and contextual fear conditioning [32] as well as the return of fear (Lonsdorf, Haaker & Kalisch, under review) as well as by others [37]–[39].

In our extended 3-day version of this paradigm, three pictures of rooms, presented for 60 s, served as context CSs (“CXT”) during each of which one of three discrete visual symbols (cue CSs, “Cue”) were intermittently shown (see **Fig. 1** and sections on “stimulus material”, “trial structure” and “procedure” for details). Three conditions (unpredictable, predictable, safe) were realized through different predictability of the US. In the *unpredictable condition*, the cue (UCue) did not signal the US, making the context itself (UCXT) the best US predictor (context conditioning). Cue conditioning on the other hand should occur in the *predictable condition* where the cue (PCue) always co-terminated with the US, making the cue a better US predictor than the context (PCXT). In a *safe condition*, providing control stimuli SCue and SCXT, no US was given. Of note, in contrast to one version of the NPU-threat test [33], during conditioning two presentations of the UCue were accompanied with an US to avoid that the UCue acquires safety-signal properties [32]. Thus, to avoid that the UCue acquired safety signal properties, two USs in total were applied during UCue presentations (1 s after cue onset; never during the first 2 two UCue presentations) and to avoid that the onset of the UCXT acquires safety signal properties a US occurred 2 s after UCXT onset once (never in the first block). This three “special cases” (2 UCue and one UCXT onset) was omitted from SCRs scoring.

Following conditioning (day 1) and extinction (day 2), context and cue CSs were again presented a week later in two memory expression tests (day 8). Here, after initial CSs presentations (“Test 1”), participants received a series of three unsignaled USs followed by additional CS presentations (“Test 2”), to induce reinstatement of conditioned responding [5].

We thus expected fear expression to prevail after the reinstatement procedure (Test 2), as evident from a relative increase of conditioned responding from Test 1 to Test 2.

### Participants

99 Participants aged 20–46 (age mean±s.e.m. 26.1±0.5, 70 females) years were recruited via advertisements at the local universities as well as via a website. Exclusion criteria were any known current or prior neurological or psychiatric disorders, use of prescription drugs within the past two months or use of non-prescription drugs during the last two weeks preceding the experiment as well as the use of illegal drugs of abuse. Trait anxiety scores [40] ranged from 23 to 52 (mean 33.1±0.5), in agreement with values from a German norm population [41].

Six participants were excluded from the whole study during day 1 (technical difficulties on day 1: N = 3; dropout on day 1: N = 3). Leaving 93 participants for analysis. Additional five participants did not return on day 8 and were thus excluded from day-8 analyses.

### Ethics statement

All participants provided written informed consent. The study was approved by the ethics committee of the Ärztekammer (General Medical Council) Hamburg (PV3378).

### Stimulus material

#### Conditioned stimuli

Three background pictures of rooms were used as context CSs (CXT) and three geometric symbols (triangle, circle, star) served as cue CSs (Cue) (**Fig. 1B–D**). A black screen with a white fixation cross was shown during the inter-trial intervals (ITIs). The visual stimulus material was presented on a computer screen (24′′; 1920×1200 pixels). Stimuli were presented using Presentation® software (NeuroBehavioral Systems, Albany California, USA).

#### Unconditioned stimulus

An electrotactile stimulus consisting of a train of 3 square-wave pulses of 2 ms duration each (interval 50 ms) served as the US. The US was delivered through a surface electrode with platinum pin (Specialty Developments, Bexley, UK) on the right dorsal hand using a DS7A electrical stimulator (Digitimer, Welwyn Garden City, UK).

#### Startle probe

A 95dB[A] burst of white noise was used as startle probe and presented binaurally via headphones (Sennheiser, Wedemark, Germany).

### Trial structure

Stimuli were presented in trials that corresponded to the continuous presentation of one of the CXTs and lasted for 60 s. ITI duration was 6–8 s with a mean of 7 s. During a trial, the corresponding cue was presented twice for 5 s during fixed time windows (with an onset at 16–18 s and 39–41 s after trial onset) (**Fig. 1B–D**). Assignments of the contextual and cue CS to three pairs corresponding to the unpredictable (UCXT, UCue), the predictable (PCXT, PCue) and the safe condition (SCXT, SCue), were consistent across the experiment and across days, but varied between participants. While stimulus combinations in the unpredictable and predictable conditions were counter-balanced across participants, the stimuli in the safe condition were always the ones shown in **Fig. 1D**. On each day, trials were presented in either of four pseudo-randomized orders, whereof two started with a predictable and two with an unpredictable condition (balanced across participants). There were no more than two consecutive trials belonging to the same condition. Trials were always grouped in blocks of 9 (3 of each condition) that were separated by subjective fear ratings (see below).

In each block, 32 startle probes were presented whereof 3–4 probes per condition were presented during Cue presentations and 5–6 probes per condition were presented outside Cue presentations (when only the CXT picture was visible), resulting in a total of 17, 11 and 22 startle probes to CXT CSs (on day 1, day 2 and day 3, respectively) and in 10, 7 and 14 startle probes to cue CSs (on day 1, day 2 and day 3, respectively). In addition, 5 presentations of ITI startle probes were included in every block resulting in a total of 15, 10 and 20 ITI startle probes (on day 1, day 2 and day 3, respectively).

Startle probes occurred 2.5 or 3.5 s and between 3.5 s and 7.5 s post-stimulus onset during Cue and Context presentations respectively. ITI startle probes occurred 3 s or 4 s post-ITI onset. It was made sure, that the startle probes did not interfere with the US administrations (in particular during the presentation of the UCXT; startle probes always appeared before possible US administrations to maintain maximal comparability to the startle probes presented during PCXTs).

### Procedure

Day 1 (Conditioning), day 2 (extinction, approx. 24 h after conditioning) and day 8 (Test 1, Test 2, 6–8 days after conditioning) took place in the psychophysiological laboratory, where up to four participants were recorded simultaneously. Participants were seated in shielded compartments and could not see each other during the experiment (see **Fig. 1A**).

#### Day 1 (conditioning)

The procedure included attachment of recording and stimulation electrodes as well as individual calibration of the US intensity to a level of maximum tolerable pain (range 0.7–85 mA, mean 7.4±0.9 mA). Participants were asked to rate the painfulness of the US between 0 (“I feel nothing”) and 10 (“maximally unpleasant”) (final rating: range 3–10, mean 7.4±0.1). While calibration was being conducted for one participant, the other participants listened to loud music via headphones. During a habituation phase, each of the three trial types was presented in a shortened exemplary version without any US. Participants were also familiarized with the fear rating scales and the use of the keypad. Conditioning consisted of 27 trials in 3 blocks (total of 9 trials per condition). In the unpredictable condition, one, two, or three USs per trial (with a mean of two) were randomly administered in fixed time windows (with onsets between 8–10 s, 30–32 s or 54–56 s after trial onset). To avoid that the UCue acquired safety signal properties, two USs in total were applied during UCue presentations (1 s after cue onset). In the predictable condition, the PCue was always paired with a US occurring 4.8 s after cue onset (100% PCue reinforcement). Thus, in both conditions, the same total number of USs was administered. In the safe condition, no US ever occurred. Participants were not informed about the conditioning contingencies or the learning element beforehand.

At the end of the experiment, CS-US contingency awareness was assessed using a semi-structured interview [42], based on which participants were classified as aware and unaware. After conditioning, 74 participants were classified as aware and 19 were classified as unaware, suggesting globally successful conditioning.

#### Day 2 (extinction)

Approximately 24 hours after conditioning, participants returned to the laboratory. Stimulation and recording electrodes were attached at the same positions as the day before, without renewed US intensity calibration. During the experiment, 18 trials were presented in 2 blocks (total of 6 trials per condition). No US was administered. Participants were not informed beforehand about any change in CS-US contingencies as compared to the previous day.

#### Day 8 (Test 1 & Test 2)

Participants returned to the laboratory and stimulation and recording electrodes were again attached. There was no additional US calibration. A recall test (Test 1) consisted of 18 unreinforced trials in 2 blocks (total of 6 trials per condition) and was followed by the presentation of a grey screen [20], [22], [26]. Five seconds after onset of the grey screen, 3 unsignaled reinstatement-USs were administered (interval 5 s). The intensity used was that chosen on day 1. Reinstatement of fear is thought to require presentation of the reinstatement-USs in a context identical to the test context [5], [15]. Reinstatement-USs were here presented in the same global context (that is the psychophysiological laboratory) as the CSs during Test 1 and Test 2, but not while any of the experimental context CSs were present. This was done to avoid re-acquisition of any of the context CSs. However, the grey background on which the reinstatement-USs were presented [20], [22], [26] also introduced a physical distinction from the tests.

Two minutes after the last US, a reinstatement test (Test 2, corresponding to Test 1) was conducted. The interval between reinstatement-USs and Test 2 served to reduce potential non-associative effects of the USs on subsequent CRs [11]. The very first startle probe following the reinstatement manipulation was administered during the first ITI (meaning before presentation of any of the CSs) for all participants to capture possible sensitization effects.

### Behavioral measures

#### Fear ratings

At the beginning of each experimental phase as well as after every trial block, participants were asked to rate each CS with respect to the fear/stress/tension that was elicited when they last saw it. Ratings were performed on a computerized Visual Analogue Scale [VAS, 0 (none) – 100 (maximal)], using the keyboard with the right hand. Selected rating values had to be confirmed by a key press and were otherwise treated as missing data. Participants were excluded from the analyses (day-wise) if less than one third of all data points (single trial reactions) were valid (meaning if more than 2/3 of the datapoints were missing) [excluded participants: N(day 1) = 2, N(day 2) = 8, N(day 3) = 6]. See tables 1 and 2 for the exact N included in the different analyses. Ratings prior to the first experimental phase (conditioning, day 1) were not included in the analyses.

**Table 1:** Results for the contextual CSs.

Measure	Phase	N	df	F	p	Eta^2^	Contrasts
**Ratings**	**C**	91	2,180	166.67	<0.001	0.65	1
	**E**	85	2,168	21.37	<0.001	0.20	1
	**T1**	76	2,150	22.14	<0.001	0.22	2
	**T2**	81	2,160	5.77	0.008	0.07	2
	T2>T1	81	1,79	4.392	0.036	0.06	—
**FPS**	**C**	66	3,195	36.60	<0.001	0.36	6
(+ITI)	**E**	46	3,135	6.29	<0.001	0.12	4
	**T1**	49	3,144	11.374	<0.001	0.19	5
	**T2**	49	3,144	1.315	0.272		—
	T2>T1	49	1,48	13.662	<0.001	0.22	—
**SCR**	**C**	65	2,128	13.00	<0.001	0.17	2
	**E**	68	2,134	<1	0.667	—	—
	**T1**	66	2,130	5.663	0.008	0.08	2
	**T2**	65	2,128	<1	0.629		—
	T2>T1	64	1,63	3.793	0.056	0.06	—

Main effects of stimulus (UCXT, PCXT, SCXT) in the during conditioning (C, day 1), extinction (E, day 2) and the memory tests on day 8 before (Test 1, T1) and after reinstatement (Test 2, T2). Main effects of time are given to index changes from T1 to T2 (T2>T1, indicative of a generalized reinstatement).

1 =  all contextual CSs differ significantly from each other (UCXT>PCXT>SCXT).

2 =  UCXT and PCXT do not differ significantly from each other but both differ significantly from the SCXT.

3 =  UCXT differs significantly from all other contextual CSs, the ITI differs significantly from all contextual CSs.

4 =  all contextual CSs differ significantly from the ITI, but not from each other.

5 =  UCXT and PCXT do not differ significantly from each other, but both differ significantly from the SCXT, the ITI differs from UCXT and PCXT.

6 =  UCTX differs significantly from all other contextual CSs, the ITI differs significantly from all other contexts.

**Table 2:** Results for the cued CSs.

Measure	Phase	N	df	F	p	Eta^2^	Contrasts
**Ratings**	**C**	91	2,180	130.03	<0.001	0.59	1
	**E**	86	2,170	27.37	<0.001	0.24	2
	**T1**	79	2,156	17.07	<0.001	0.18	3
	**T2**	73	2,144	1.88	0.173		—
	T2>T1	73	1,70	1.64	0.207		—
**FPS**	**C**	66	3,195	52.33	<0.001	0.45	2
(+ITI)	**E**	47	3,138	10.04	<0.001	0.18	5
	**T1**	49	3,144	10.172	<0.001	0.18	5
	**T2**	49	3,144	<1	0.974		—
	T2>T1	49	1,48	<1	0.994		—
**SCR**	**C**	65	2,130	33.59	<0.001	0.34	1
	**E**	68	2,134	4.20	0.018	0.06	2
	**T1**	66	2,130	3.348	0.049	0.05	2
	**T2**	65	2,128	2.2175	0.119		—
	T2>T1	64	1,63	7.980	0.006	0.11	—

Main effects of stimulus (UCue, PCue, SCue) in the during conditioning (C, day 1), extinction (E, day 2) and the memory tests on day 8 before (Test 1, T1) and after reinstatement (Test 2, T2). Main effects of time are given to index changes from T1 to T2 (T2>T1, indicative of a generalized reinstatement).

1 =  all cued CSs differ significantly from each other (PCue>UCue>SCue).

2 =  PCue and UCue do not differ significantly from each other but both differ significantly (>) from SCue and ITI.

3 =  PCue and UCue differ significantly from SCue, PCue and UCue differ trendwise from each other.

5 =  all cued CSs differ significantly from the ITI, but not from each other.

#### Skin conductance

Skin conductance was measured via self-adhesive Ag/AgCl electrodes placed on the palmar side of the left hand on the distal and proximal hypothenar. Data were recorded with a BIOPAC MP-100 amplifier (BIOPAC Systems Inc, Goleta, California, USA) with AcqKnowledge 4 software. Data were down-sampled to 10 Hz and phasic skin conductance responses (SCRs) to the onsets of CXT or cue CS were manually scored off-line using a custom-made computer program. SCR amplitudes (in µS) were scored as the largest response occurring 0.9 to 4.0 s after CXT or Cue onset [43]. Non- reactions were scored as zero and trials with obvious electrode artefacts were scored as missings. As before [32], we did not analyze the rest of the CXT presentation periods as in the predictable condition these are confounded by US reactions. Separately for the three experimental days, logarithms were computed for all values, to normalize the distribution [44], and these log values were range-corrected (SCR/SCR_max CS [day]_) to account for inter-individual variability [45]. SCR measurements that showed recording artefacts or excessive baseline activity were discarded and treated as missing data. SCR data from a limited number of participants had insufficient data quality (as judged by two researches; due to technical difficulties during recording or less than one third valid single trial reactions) and were thus excluded (day-wise) from the analyses [N(day 1) = 26, N(day 2) = 25, N(day 8) = 22]. See tables 1 and 2 for the exact N included in the different analyses.

**Figure 2:**
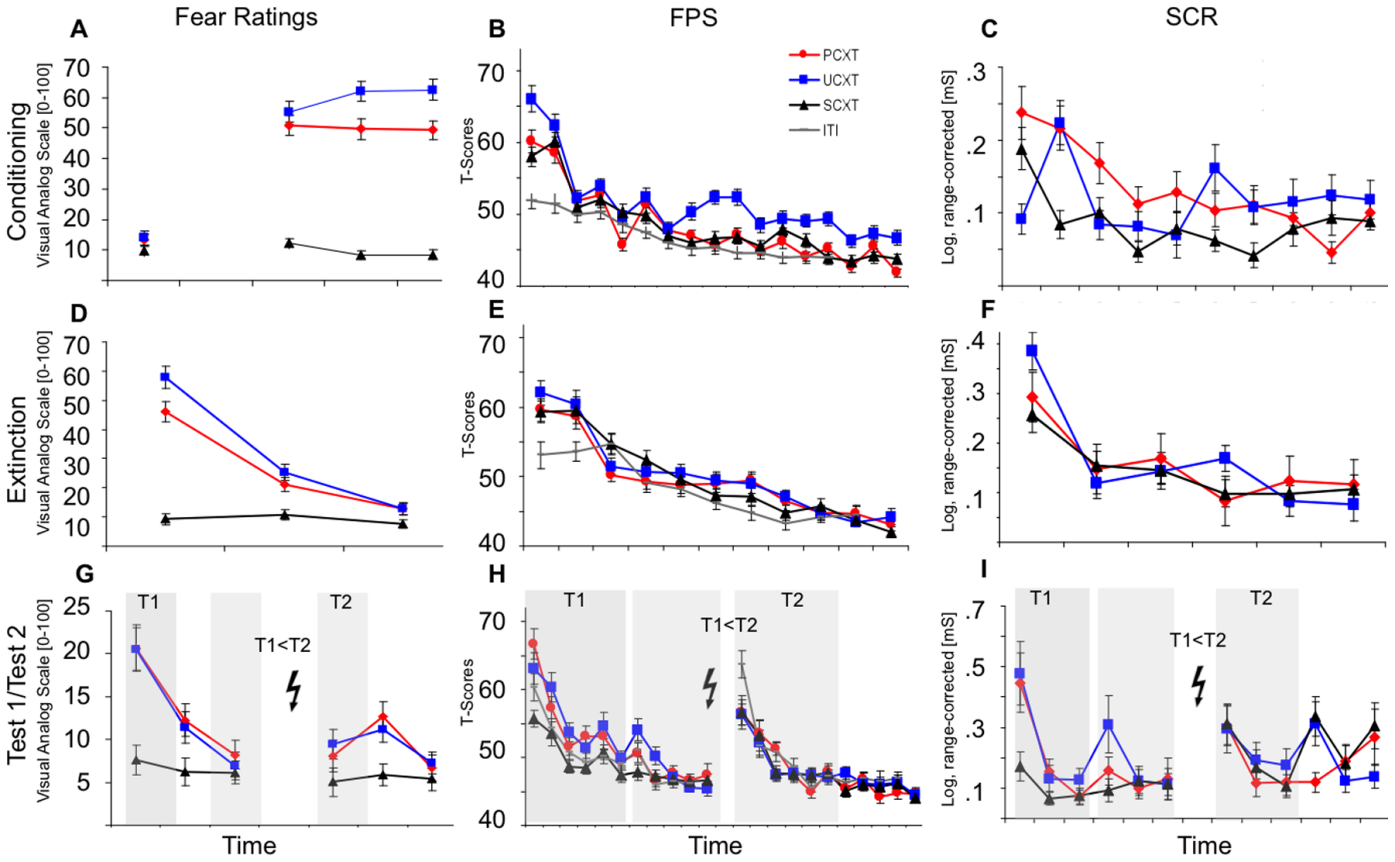
Fear Ratings (A,D,G), FPS (B,E,H) and SCRs (C,F,I) during Conditioning (A,B,C), Extinction (D,E,F) and Test1/Test2 (G,H,I) for contextual CSs (single-trials). Grey shades for day 8 represent trial blocks that were used for statistical analyses. Data show mean±s.e.m., SCRs are logarithmized and range-corrected, FPS represent T-scores. PCXT =  predictable context, UCXT =  unpredictable context, SCXT =  safe context; Bolt denotes US.

SCRs were averaged over blocks of 3 (context conditioning) or 6 (cue conditioning) trials, resulting in 3 blocks on day 1, 2 blocks on day 2 and 2 blocks per Test on day 8 (as in **Fig. 2**).

#### Fear potentiated startle

Startle reactions were measured by recording electromyographic (EMG) activity over the orbicularis oculi muscle beneath the left eye using miniature Ag/AgCl electrodes. The EMG signal was amplified and filtered through a BIOPAC MP-100 amplifier (BIOPAC Systems Inc, Goleta, California, USA) and recorded with AcqKnowledge 4 software. Data were down-sampled to 100 Hz and manually scored off-line using a custom-made computer program. The magnitude of the startle eyeblink (in microvolts) was measured from onset to peak, as described previously [46]. Blink magnitudes were normalized using z-standardization and converted to T-scores to ensure that all participants contributed equally to the group mean. FPS data from a limited number of participants had insufficient data quality (as judged by two researches due to technical difficulties during recording or less than one third valid single trial reactions) and were thus excluded (day-wise) from the analyses [N(day 1) = 27, N(day 2) = 41, N(day 8) = 28]. Startle reactions were scored as missing if a blink occurred immediately before startle probe administration or due to obvious electrode artefacts. See tables 1 and 2 for the exact N included in the different analyses.

#### Data analysis

Behavioral data were transformed and analyzed (using SPSS 18 for Windows) separately for the three experimental days [47] and no comparisons between days were performed. For fear ratings, repeated-measures ANOVAs with stimulus (3) as the within-subject variable were applied for days 1 and 2. For SCRs and FPS repeated-measures ANOVAs including the effect of time (block) were calculated. In contrast to fear ratings where only few data points throughout the experimental sessions exist, the factor block was included for SCR and FPS analyses to provide a more fine-grained picture of the learning curves (indicated by interactions of stimulus with time). In addition, in the FPS analysis the ITI responses were included to allow for analysis of generalized startle potentiation to all CSs as compared to this baseline condition.

For day 8, the different ANOVAs testing memory expression before (Test 1) as well as after (Test 2) reinstatement were restricted to stimulus effects (Ratings, SCR: 3, FPS: 4) in the first blocks of each test, to account for on-line extinction. A potential enhanced fear memory expression after versus before reinstatement (Test 2>Test 1) was assessed using stimulus (Ratings, SCR: 3, FPS: 4) x time (2) repeated-measures ANOVAs on the last block before and the first block after reinstatement. Effects of interest for the reinstatement test were a main effect of time (indicative of generalized reinstatement) as well as a time x stimulus interaction (indicative of CS specific reinstatement). An α-level of p<0.05 was considered significant and Greenhouse-Geisser correction was applied if necessary.

## Results

### Context conditioning

#### Fear ratings

Fear ratings showed robust conditioning effects as indicated by significant main effects of stimulus that were maintained throughout extinction and the memory tests before (Test 1) and after (Test 2) reinstatement (all ps<0.008, see **Figure 2 A,D,G** and **Table 1** for statistical details). Participants reported most fear to the UCXT and showed significant discrimination (UCXT>PCXT>SCXT) between all three contextual CSs during conditioning and extinction. During Test 1 and Test 2, no discrimination between the UCXT and the PCXT was observed while both were rated significantly higher than the SCXT (see “Contrasts” in **Table 1**).

The reinstatement manipulation significantly enhanced fear ratings in general, as indicated by a main effect of time (T2>T1; see **Figure 2G** and **Table 1**) but this effect was driven in particular by the UCXT, as indicated by a significant stimulus x time interaction, F(2,158) = 5.04, p = 0.016, Eta^2^ = 0.060.

#### FPS

Robust conditioning effects were also evident in FPS on day 1 and were maintained during extinction and Test 1 (all ps<0.001), but not Test 2, (see **Figure 2 B,E,H** and **Table 1**). On day 1, the UCXT elicited higher startle responses than any other contextual CSs and the ITI (ps<0.001), while the ITI elicited lower responses than any contextual CS (all ps<0.001). During extinction this discrimination between the contextual CSs and the ITI was maintained (all ps<0.013) while all contextual CSs elicited comparable responses (ps>0.1, see **Figure 2**). During the memory test on day 8 (Test 1), both the UCXT and the PCXT elicited significantly higher responses than the SCXT and the ITI (all ps<0.002), both of which elicited comparable responses (p>0.8).

As for fear ratings, reinstatement effects manifested as a significant main effect of time (T2>T1, generalized reinstatement see **Figure 2H** and **Table 1**) as well as a weak stimulus x time interaction, F(3,144) = 2.16, p = 0.097, Eta^2^ = 0.043, driven by a slightly more pronounced reinstatement effect for the SCXT as compared to the ITI and the UCXT (both ps<0.045). Note that an ITI startle probe represented the very first stimulus after the reinstatement manipulation for all participants irrespective of stimulus-sequence (see methods). This generalized enhancement of responding to all contextual CSs (T2>T1) in combination with the absence of a significant stimulus effect on Test 2 indicates a generalized (meaning non-differential) effect of the reinstatement manipulation.

#### SCRs

Similarly, SCRs revealed main effects of stimulus only during conditioning and Test 1 (p<0.008, see **Figure 2C,F,I** and **Table 1**). Responses during these phases were comparable for UCXT and PCXT (all ps>0.6) and significantly lower for SCXT (all ps<0.004). In addition, there was a stimulus x time interaction during conditioning, F(4,256) = 3.75, p = 0.011, Eta^2^ = 0.06, as a consequence of decreasing SCRs from the first to the second and from the first to the third block for the PCXT and maintained high reactivity to the UCXT (both ps<0.05) and maintained low reactivity to the SCXT (both ps<0.09; and from the second to the third block, p = 0.01).

Furthermore, on day 8, there was a strong trend for a generalized reinstatement effect (main effect of time) in absence of an interaction with stimulus, again as for FPS, suggesting a generalized non-differential reinstatement of fear. This is supported by the absence of a significant stimulus effect on Test 2 (as for FPS) as a result of non-differentially increased responding to all contextual CSs following reinstatement.

In sum, all three dependent measurements revealed robust context conditioning effects with the highest fear- (FPS, Ratings) and arousal (SCR) related responses to the UCXT. During extinction, the responses to both shock-associated contexts (UCXT and PCXT) elicited comparable responses in subjective ratings and fear-specific responses (FPS). The same pattern was still present during the delayed memory test (Test 1), but here also for the arousal-related measurement (SCRs).

Of note, reinstatement effects for contextual CSs were evident in all dependent measures as a significant generalized enhancement in subjective ratings and fear-specific measures (FPS) and a trend-wise generalized enhancement of arousal (SCRs). In addition, a specific increase in explicit fear ratings was observed for the UCXT indicating differential return of fear.

### Cue conditioning

#### Fear ratings

**Figure 3:**
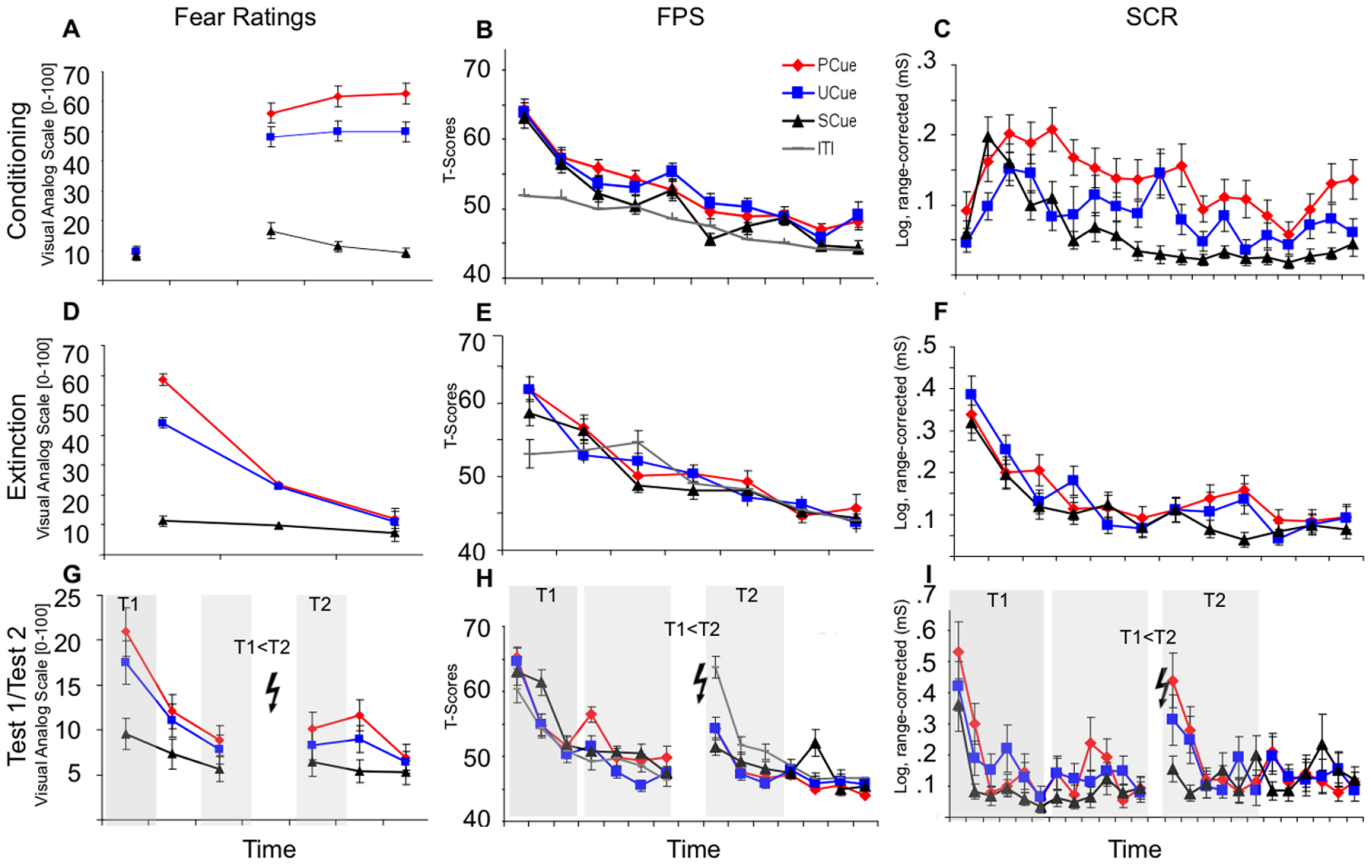
Fear Ratings (A,D,G), FPS (B,E,H) and SCRs (C,F,I) during Conditioning (A,B,C), Extinction (D,E,F) and Test1/Test2 (G,H,I) for cued CSs (single-trials). Grey shades for day 8 represent trial blocks that were used for statistical analyses. Data show mean±s.e.m., SCRs are logarithmized and range-corrected, FPS represent T-scores. PCue =  predictable cue, UCue =  unpredictable cue, SCue =  safe cue; Bolt denotes US.

Fear Ratings showed robust conditioning effects as indicated by a significant main effect of stimulus that was maintained throughout extinction and the memory test before reinstatement (Test 1, all ps<0.001) but not during Test 2 after reinstatement (see **Figure 3A,D,G** **Table 1** for statistical details).

Participants reported most fear to the PCue and showed significant discrimination (PCue>UCue>SCue [all ps<0.001)]) between all three cued CSs during conditioning. As for contextual CSs, during extinction and Test 1, ratings for PCue and UCue were comparable (p>0.5), while both differed significantly from the SCue (all ps<0.06; UCue vs. SCue at trend-level p = 0.053 during Test 1).

In contrast to the ratings for contextual CSs, neither differential nor generalized reinstatement effects were observed, as indicated by the absence of a main effect of, or an interaction with time (see **Figure 3G** and **Table 1**).

#### FPS

Conditioning effects were evident in FPS on day 1 (see **Figure 3B**) with comparable reactions elicited to probes presented during the PCue and the UCue (p>0.3), which in turn were significantly higher than those elicited during the SCue (all ps<0.002). In addition, all responses elicited during the cued CSs were potentiated against the ITI (ps<0.001). During extinction and Test 1 (see **Figure 3E,H** **)** the significant stimulus effect was maintained but resulted from potentiated responses to all cued CSs in comparison to the ITI (see contrasts, **Table 1**).

In analogy to fear ratings, neither a differential (stimulus x time interaction, p = 0.11) nor a generalized (main effect of time, see **Figure 3H** **and** **Table 1**) reinstatement effect was observed and all stimuli elicited comparable responses during Test 2. Note that an ITI startle probe represented the very first stimulus after the reinstatement manipulation for all participants irrespective of stimulus-sequence (see methods) explaining the high startle amplitude to the very first probe following reinstatement, that was not significant in the block analyses.

#### SCRs

Analysis of SCRs revealed a main effect of stimulus during conditioning that was maintained during extinction and Test 1 (all p<0.05, see **Figure 3C,F,I** and **Table 1**). During conditioning all cued CSs were discriminated based on their prediction of the US (PCue>UCue>SCue (all ps<0.002). During extinction and Test 1, the responses to the PCue and UCue were comparable (p>0.4), and both significantly higher than the responses to the SCue (both ps<0.04), in analogy to the fear ratings.

In contrast to the fear ratings and FPS, a generalized reinstatement effect was observed, indicated by a main effect of time in absence of a stimulus x time interaction (see **Figure 3I** and **Table 1**). In support of a generalized reinstatement, no stimulus effect was observed at T2.

In sum, in analogy to the contextual CSs, robust cue conditioning was indicated by a good discrimination of the different cued CSs. Again, as for contextual CSs, the responses to both shock-associated cued CSs (UCue and PCue) elicited comparable fear- and arousal related responses during extinction and the delayed memory test (Test 1).

In contrast to the contextual CSs, the effect of the reinstatement manipulation was only detectable in the arousal-related measurement (SCRs), as generally heightened responses to all cued CSs during Test 2 as compared to Test 1. The subjective ratings and fear-related measurements (FPS) however remained on the same level after as compared to before the reinstatement manipulation, which may indicate a different capacity for return of cued as compared to contextual fear in this paradigm. This different capacity matches neural correlates (fMRI) derived from a similar paradigm where we observed activation in regions of the fear network to the presentation of contextual but not cued CSs after a reinstatement manipulation (Lonsdorf, Haaker & Kalisch under review; see discussion for details).

### Additional analyses including the between-subject factor sex

Analyses that included sex as a between-subject factor were performed in order to account for described sex differences in fear conditioning and extinction [48]–[50].

#### Fear Ratings

Analyses with the inclusion of the factor sex had no impact on the results reported for any day/experimental phase in any mode of conditioning and revealed no interaction or main effects of the factor sex.

#### FPS

The inclusion of the factor sex had no major impact on the results reported and for any day/experimental phase in any mode of conditioning (cued/contextual CSs) and no significant interaction including the factor sex was observed, except for a significant sex x stimulus interaction for cued CSs on day 2, F(3,135) = 3.20, p = 0.03, Eta2 = 0.07, and during test 1 (day 8), F(3,131) = 3.13, p = 0.03, Eta2 = 0.06.

#### SCR

During conditioning on day 1, there was no effect of the factor sex on the reported results in both CS types. On day 2, the stimulus effect during extinction learning to the cued CS was no longer significant (p = 0.15). On both days, there was no significant main effect or interaction with the factor sex. The analyses of Test 1 changed (from significance) to a trend-wise significant stimulus effect for the cued CSs [F(2,128) = 3.024; p = 0.064; eta^2^ = 0.045] and contextual CS [F(2,128) = 2.877; p = 0.074; eta^2^ = 0.043] after inclusion of the factor sex. In addition, sex had a main effect during Test 2 for cued CSs [F(1,63) = 16.599; p<0.001; eta^2^ = 0.209] and on the reinstatement test [(T2>T1), F(1,63) = 16.599; p<0.001; eta^2^ = 0.209]. Further, there was a significant interaction between time and sex [F(1,62) = 4.820; p = 0.032; eta^2^ = 0.072] during the reinstatement test (T2>T1) for the cued CS.

## Discussion

The aim of this present study was to investigate delayed recall and reinstatement of cued and contextual conditioned responses using a multidimensional approach and a multiple-day paradigm that allows for consolidation to occur in between the experimental phases.

First, our findings are in line with animal work that demonstrated the persistence of conditioning and extinction memory traces over a time delay [13], [51], [52] as we observe discriminative responding at a one-week delayed recall test (Test 1) in all measurement modalities (subjective fear ratings, FPS, SCRs) to both contextual and cued CSs. For contextual CSs, responses to both shock-associated contexts were enhanced as compared to the control condition (safe context) while, for cued CSs, this was the case for fear ratings and SCRs, whereas FPS reactions to all cued CSs were potentiated against the ITI.

Second, we observed that reinstated fear responses were qualitatively different between the different dependent variables (subjective fear ratings, FPS, SCRs) as well as between cued and contextual CSs. Generally, reinstatement-induced enhanced physiological responding was transient and obvious only in the two first trials post-reinstatement.

While only the arousal-related measurement (SCRs) showed increasing reactions following reinstatement (Test 2 > Test 1) to the cued CSs, no evidence of reinstatement was observed for the subjective ratings and fear-related measurements (FPS). In contrast, for contextual CSs, reinstatement was evident as significantly elevated fear related measurements (fear ratings, FPS) and trend-wise enhanced SCRs to all contextual CSs (generalized non-differential reinstatement). In addition, fear ratings were also specifically elevated to the UCXT following reinstatement (differential reinstatement).

Generally enhanced responses following the reinstatement manipulation may call into question whether these can in fact be attributed to association-based processes or if they rather represent sensitization. The observation of non-differential (generalized) return of fear is not restricted to reinstatement procedures but is also evident in renewal [53] and spontaneous recovery [54]. As discussed by Vervliet and colleagues [53], this does not preclude a true return of the CR to the CS+ but may reflect associative learning processes related to the CS-, which would suggest that the CS- is not a pure control stimulus.

In our data, enhanced ITI startle responses observed immediately after the reinstatement manipulation may be interpreted as evidence that sensitisation effects may (at least partly) underlie the enhanced responses following reinstatement. However, the differential reinstatement effect observed in fear ratings renders an exclusive effect of sensitization rather implausible.

Even more convincingly, sensitization would be expected to result in generally enhanced reactions to all CSs (cued and contextual CSs). We however observe no reinstatement effect to cued CSs in both subjective ratings and the fear-related modality (fear ratings), which strongly speaks in favour of association-based underpinnings of the observed reinstatement effects. In addition, the neural correlates of reinstated cued and contextual fear observed in an identical experimental paradigm using fMRI (Lonsdorf, Haaker, Kalisch, under review) further support association-based processes: We observed in two independent samples responses in the vmPFC to the PCue (PCue>SCue) following reinstatement, a region that has been implicated in extinction memory recall in previous studies [55], [56], a finding that nicely fits with the lack of reinstatement in fear-related modalities and subjective fear ratings in our present study. Contextual CSs (UCXT>SCXT) after reinstatement in turn, were associated with amygdala activity, mirroring the present findings of returning fear as measured by FPS and fear ratings. Notably, a recent review highlights the role of the amygdala in contextual processing [57] and animal work has also implicated the amygdala in reinstatement of fear [16], [58].

The disparate return of fear towards cued and contextual CSs in our paradigm followed unsignalled presentations of the US while participants viewed a grey screen, as in previous studies of reinstatement in humans [20], [22], [26]. This background screen was different from the background rooms (context CSs) in which the cue CSs were embedded throughout the experiment. Studies in rodents [5], [10] and humans [18], [19] imply that the reinstatement of a cued CR is attenuated if the context in which the reinstatement-USs are administered differs from the context of CS presentation during the test [15]. This raises a principle problem when trying to study the reinstatement of contextual fear, which necessitates to establish a “context for the context” or superordinate context. We here hoped that the unsignalled reinstatement-USs would imbue the general test situation with a sense of danger and enhanced US expectancy and therefore establish it as a superordinate, US-associated context that facilitates the return of contextual fear by gating the retrieval of the initial context CS-US association [5], [53].

Our results indicate that this hypothetical superordinate context-US association exerted a stronger effect on the retrieval of contextual as opposed to cued CS-US associations, indicated by a pronounced, albeit generalized, return of contextual fear in FPS and differential return of fear in fear ratings, whereas the reinstatement of cued fear was absent in these measurements. This would imply that the superordinate context only governed the reinstatement of fear to the rooms (context CSs) but was not perceived as a relevant context for the symbols (cue CSs). Reinstatement of fear to the latter was presumably under the control of the rooms (context CSs) in which they were directly embedded.

This hierarchical order of contextual gating in reinstatement would be in line with demonstrations of contextual dependence of reinstatement in rodents and humans: reinstatement is stronger if the reinstatement USs are administered in the context that directly surrounds the CSs during test [10], [18], [19], [15].

An alternative explanation for the disparate return of cued and contextual fear might be that the expression of fear to contextual CSs may be the appropriate defensive response (acquired through *unpredictable* US administrations during day 1) after experiencing *unpredictable* USs during reinstatement. Of note, participants could in principle learn to anticipate the temporal sequence of US administration in the unpredictable condition on day 1, but the results of the awareness-interview do not point towards this [37]. Thus, similarities in unpredictability of the US between contextual fear learning on day 1 and the reinstatement manipulation may selectively promote the return of fear to contextual CSs. Mechanistically speaking, the US re-confrontation during reinstatement is assumed to re-activate the CS-US association generated during conditioning. In our paradigm, two different CS-US associations, a cued CS-US_predictable_ and a contextual CS-US_unpredictable_ association, might have been acquired during cued and contextual conditioning respectively. Consequently, the re-confrontation with unsignalled and thus unpredictable USs during the reinstatement manipulation might result in a predominant retrieval of the contextual CS-US_unpredictable_ association, resulting in the expression of contextual fear.

Thus, even if prior studies of reinstatement in humans demonstrated return of fear to cued CSs after administration of unpredictable reinstatement-USs [18], [20]–[24], these results are based on paradigms of cued fear learning where the US was exclusively associated with a cued CS.

Our paradigm in turn, employed for the first time a more complex experimental situation and tested the expression of the CR to cued and contextual CSs after reinstatement.

Future research may profit from the use of such complex paradigms which may be suitable to characterize individual differences in the return of fear (e.g. generalized vs. differential return of fear), preferably combined with multimodal assessment of the CR.

There are some possible limitations of our work, that deserve to be mentioned. First, the intensity of the reinstatement USs used was identical to the intensity during conditioning. It can however not be excluded that participants experienced the intensity differently during day 8 and future studies should employ additional ratings of reinstatement US aversiveness in order to rule out that this affects results. Second, the number of unaware participants did not allow us to statistically compare aware and unaware participants with respect to the performance in our study. Future studies may experimentally manipulate contingency awareness to study effects on the return of fear following reinstatement. Third, due to the simultaneous acquisition of data of up to 4 participants, the drop-out rates in our psychophysiological measures are somewhat higher than usual. Importantly however, dropouts for FPS and SCRs did not differ from non-dropouts in STAI trait scores, both F<1, both p>0.3, suggesting that this did not produce a systematic bias in our sample. It can however not finally be excluded that drop-outs might have affected different measurements differentially.

The return of fear after reinstatement is likely to depend on a variety of variables like the experimental design (e.g. time-gaps between experimental phases, context of reinstatement - US administration) and dependent measurements (arousal or fear-specific). Many questions with respect to the critical determinants of reinstatement in humans remain to date and future research requires more systematic investigations.
